# Rapid Multi-Damage Identification for Health Monitoring of Laminated Composites Using Piezoelectric Wafer Sensor Arrays

**DOI:** 10.3390/s16050638

**Published:** 2016-05-04

**Authors:** Liang Si, Qian Wang

**Affiliations:** 1 Institute of Lightweight Structures, Faculty of Mechanical Engineering, Technische Universität München, Garching 85748, Germany; 2 Department of Electrical and Computer Engineering, Technische Universität München, Munich 80333, Germany; wang@lrz.tu-muenchen.de

**Keywords:** multi-damage identification, structural health monitoring, Lamb wave, delamination, piezoelectric wafer sensor array, mode decomposition, Hilbert spectral analysis, energy density, phase shift

## Abstract

Through the use of the wave reflection from any damage in a structure, a Hilbert spectral analysis-based rapid multi-damage identification (HSA-RMDI) technique with piezoelectric wafer sensor arrays (PWSA) is developed to monitor and identify the presence, location and severity of damage in carbon fiber composite structures. The capability of the rapid multi-damage identification technique to extract and estimate hidden significant information from the collected data and to provide a high-resolution energy-time spectrum can be employed to successfully interpret the Lamb waves interactions with single/multiple damage. Nevertheless, to accomplish the precise positioning and effective quantification of multiple damage in a composite structure, two functional metrics from the RMDI technique are proposed and used in damage identification, which are the energy density metric and the energy time-phase shift metric. In the designed damage experimental tests, invisible damage to the naked eyes, especially delaminations, were detected in the leftward propagating waves as well as in the selected sensor responses, where the time-phase shift spectra could locate the multiple damage whereas the energy density spectra were used to quantify the multiple damage. The increasing damage was shown to follow a linear trend calculated by the RMDI technique. All damage cases considered showed completely the developed RMDI technique potential as an effective online damage inspection and assessment tool.

## 1. Introduction

A reliable, effective and autonomous damage assessment technique for composite structures will be a very important factor in smart aerospace system design in the future. Damage generated in aerospace composite structures due to the various events and reasons may not be visible to surface inspection but still can cause significant loss of structural integrity. Especially, the delamination damage type arises in laminated composites due to a variety of reasons. Flaws in the manufacturing process, debonding or even impact damage can cause delaminations between the plies of laminated composites. Though not visible in the surface of composites, a delamination is still able to result in a loss in structural stiffness and strength, and further may conduct to catastrophic failure. Therefore, this provides the motivation for this study. 

With the use of the acousto-ultrasonic method, a methodology to interpret the altering results of probing waveforms needs to be proposed and developed since the modulated Lamb waves are excited from an actuator. In order to locate and quantify structural damage, several methods have been proposed to enhance and convince the interpretation of the Lamb wave signals measured by sensors. The damage inspection (DI) methods are mostly based on changes in wave attenuations, for instance, Ihn and Chang [1] proposed the attenuation index (AI) map to serve for each diagnostic path, and the AI was used in terms of a measure of a signal energy depletion compared with the baseline signal. The DI methods are generally classified into five major approaches that are: (1) wavelets, as reported by Qiu *et al.* [2], (2) the spectral finite element analysis methods of Yu and Giurgiutiu [3] and Kudela and Ostachowicz [4], (3) the time-frequency analysis of Liu *et al.* [5], (4) statistical analysis methods such as those of Qiu *et al.* [6] and Liu *et al.* [7], and (5) the information of time of arrival/flight (TOA/TOF) from wave reflections/scatterings described by Zhu *et al.* [8]. 

Nevertheless, for the time-frequency methods applied in damage inspection and evaluation, Lee and Staszewski [9] examined the power spectra of the Lamb wave data with different damage sizes for different propagation distances and discerned the variations due to damage in the dominant frequency. In the application of spectral finite element (SFE) to damage detection, Kim *et al.* [10] developed a computational model based on a spectral finite element method to simulate Lamb wave propagation with the hole and crack damage types, and verified the significant advantages of the proposed SFE method, which included the fact that the computational time for modeling Lamb wave propagation is faster than that of the FEM, and also the optimization of sensor shape, size, locations and probing waveform could be implemented. Meanwhile, the wavelet transform is also the most common time-frequency analysis tool used in the damage detection application. Okabe *et al.* [11] used the continuous wavelet transform (CWT) method to reveal the frequency dispersion of $A_{1}$ mode of propagating waves change, due to modes conversion when Lamb waves encounter a delamination. Paget *et al.* [12] utilized a wavelet method to decompose the wave responses with damage into wavelet coefficients, and validated the sensitivity of the amplitude change of the wavelet coefficients to damage severity in composite plates. As for the probability statistical analysis-based damage identification methods, Wu *et al.* [13] proposed a methodology that combined a fusion image approach and a unit weight distribution function to optimize the network of sensing paths and clarify and determine the scaling parameters for the probability based diagnostic imaging algorithm in damage identification applications. In Lamb wave reflection applications, Ip and Mai [14] designed an active diagnostic system to measure the time of arrival (TOA) of the reflected *A_0_* Lamb wave due to the through-width delamination so as to locate the delamination in fiber-reinforced plastic beam structures. From most of the aforementioned techniques, a structural health monitoring (SHM) system based on the active sensing approach with sensor network seems to be a cost-effective method for a fast and online inspection of composite structures [15]. 

In this paper, a Hilbert spectral analysis-based rapid multi-damage identification technique is proposed as a multi-damage inspection tool. Through the investigation of a series of damage experiments, the rapid multi-damage index algorithm based on multi-functional multi-metrics (MFMMs) was examined and qualified to be an effective SHM tool for locating and quantifying multiple delaminations in a two dimensional structure. During the process of multi-damage localization and damage tracking, a high-resolution energy time-phase shift spectrum provided from the novel rapid multi-damage identification technique is proposed and used with the principle and computation of wave reflections from material discontinuities; then during the process of multi-damage quantification and damage track, another energy density spectrum was developed and used to extract embedded oscillations, and to reveal hidden wave reflections information from the collected data. Overall, the rapid multi-damage identification technique based on the multi-functional multi-metrics index algorithm was applied successfully to indicate the Lamb waves interactions with a variety of damage, and this technique is developed for the modern real-time engineering inspection which is to determine the presence, location and severity of damage in carbon fiber composite structures. 

## 2. Method of Approach

The developed RMDI technique is composed of two functional modules, which are the wave velocity computation (WVC) and the damage index parameters (DIPs). In the WVC function module, the dynamics of the laminated composites is modeled by the combination of classical laminated plate theory (CLPT) [16] and finite element modelling [17], the wavenumber-frequency relationships can then be determined for a composite laminate. From the results of wave dispersion relations, the wave group velocity of the $A_{0}$ mode can be calculated out. In the DIPs function module, the multi-functional multi-metrics (referred as MFMMs) are newly proposed and developed as rapid multi-damage index parameters to analyze the variations in Hilbert time-frequency spectra and in energy time spectra due to structural flaws, and further they are used to process the sensor response data, infer the presence of damage such as delaminations, quantify the extents of delaminations, and simultaneously locate various delaminations in combination with the $A_{0}$ group velocity of the propagating waves. Moreover, the MFMMs are also enabled to trace the amount of increasing damage by their individual prediction trend curves. 

### 2.1. Determination of the Group Velocity of Propagating Waves

Lamb waves are the most widely used guided ultrasonic waves for structural damage inspection, because they can travel long distances and be applied with conformable piezoceramic transducers. Furthermore, guided Lamb waves are also able to interact with flaws in a structure due to the wave propagation properties that are highly sensitive to any discontinuity in materials. In our study, an actuation mode of sweep frequencies [60 kHz, 200 kHz] was used to generate the different low-frequency probing waves from an actuator, where the maximum frequency of 200 kHz used is much below the boundary threshold of 300 kHz between the low frequency and high frequency. Actually, the factors that are the actuation frequency, the property and structure of a composite such as the layup and thickness can determine how many Lamb wave modes exist in the composite structure. Thus for a given composite with the certain stacking sequence, fiber direction and actuation frequency, there exists a finite number of propagation modes specified by their phase velocities.

In that case, for the purpose of simplicity of analysis, it was considered that only the two fundamental modes $A_{0}$ and $S_{0}$ propagate in the tested composite panels. Since the wavelength of $S_{0}$ mode in this frequency range is above 16 mm, this mode should not be very sensitive to the defects. On the contrary, the wavelength of $A_{0}$ mode is below 10 mm (the short wavelength) in this frequency range, and thus only the $A_{0}$ mode is sensitive to the interlaminar delamination. To predict the $A_{0}$ group velocity of the propagating waves in a composite structure, the wavenumber-frequency relationships should be constructed, once the bending stiffness coefficients and the mass of the composite structure are obtained. And then, the wave group velocity can be easily deducted from the evaluated wave dispersion relations. For the low-frequency wave propagation, the CLPT theory is retained to compute the wave group velocity of the $A_{0}$ Lamb mode from the evaluated wavenumber-frequency relationships. For the high-frequency wave propagation, a finite element model constructed by Cortes [17] with the consideration of the shear deformation was used to predict the Lamb wave propagations in the CFRP panels. In our damage identification applications, the low frequency $A_{0}$ mode is more suitable to inspect and identify the inter-laminar delaminations in the tested composite panels.

In order to precisely locate multiple delaminations in a composite panel, we need to be aware of the information regarding the angular dependence of group velocity of Lamb wave. By using the two parameters of wave propagation that are the group velocity of $A_{0}$ mode and the time of flight (TOF) acquired by the developed multi-damage index algorithm detailed in Section 2.2, the locations of multiple delaminations can be inferred. Then, the relationship between group velocity and wave propagation angle *θ* is established by evaluating group velocity variation with different propagation directions. In order to obtain an actual distribution map of group velocities with regard to variable propagation angles, an experiment of the wave velocity measurements needs to be implemented. In the experiment, the propagation directions of $0^{{}^{\circ}}$ and $90^{{}^{\circ}}$ are the same as those defined in Section 3. The direction of $0^{{}^{\circ}}$ is the wave propagation direction paralleling to the longer side of the experimental specimen and pointing from the actuator toward the delamination center, and the direction of $90^{{}^{\circ}}$ is the direction of an anticlockwise rotation of $90^{{}^{\circ}}$ from the $0^{{}^{\circ}}$ direction. Meanwhile, 24 sensing nodes were laid evenly on a circle with a radius of $R_{0}$, and an actuator was arranged in the circle center to excite a series of Lamb waves. This actuator and sensor layout enables our acquisition of group velocity estimations along the direction of each sensing node. After applying a voltage signal to the actuator, Lamb waves were excited and propagate through the specimen. The response signals from the sensing nodes were also collected within the actuation frequency range of 60–200 kHz, which is consistent with that used in the damage identification tests. The distribution map of group velocity as the function of propagation angles was evaluated along all of the 24 propagation directions, which were formed through the variation of every $15^{{}^{\circ}}$ in the angular range from $0^{{}^{\circ}}$ to $360^{{}^{\circ}}$. For the *i*th propagation direction, the angle $\theta_{i}=i\times 15^{{}^{\circ}}$, *i* = 0, 1, …, 23, i∈Z. The group velocity $C_{g}\left(\theta_{i}\right)$ at the direction of the *i*th sensing node can be assessed by adopting a general strategy that is given by Equation (1):

(1) $$C_{g}\left(\theta_{i}\right)=R_{0}/TOF_{A_{0}}\left(\theta_{i}\right)$$

where $C_{g}\left(\theta_{i}\right)$ is the ratio between the radius $R_{0}$ of the circle of sensing nodes and the time of flights of the propagating wave packages measured; $TOF_{A_{0}}\left(\theta_{i}\right)$ is the time duration Δt along the direction of the *i*th sensing node for $A_{0}$ mode. It is given by $\Delta t=t_{1}-t_{0}$, where $t_{0}$ denotes the moment regarding the energy peak of the actuation from the actuator; and $t_{1}$ denotes the moment with regard to the energy peak of the actuation sensing from a sensing node *i*. $TOF_{A_{0}}\left(\theta_{i}\right)$ can be calculated through the developed damage index parameter—the energy time-phase shift metric—obtained by the rapid multi-damage index algorithm (MDIA), which is detailed in Section 2.2. The measurement of sensor signals for group velocity evaluations was repeated six times, which were performed automatically by a specific setting in the measurement equipment of acousto-ultrasonics. The group velocity profile takes the average values ${\bar{C}}_{g}\left(\theta \right)$ of group velocities at the 24 propagation angles, which is obtained from the varied excitation frequencies (60–200 kHz) repeated in six measurement times. The distribution map of the angular dependence of group velocity will be presented and detailed in Section 3. 

It is a challenge to select an effective estimation method of TOF, as the accurate extraction of the time of arrival of the instantaneous waveform is very difficult in complex and adverse environments such as recorded sensor signals having low signal-to-noise ratio [18]. A method for obtaining the energy time-phase shift metric has been proposed to attempt to overcome the difficulty. To rapidly and automatically obtain the needed TOFs, the energy-time spectra of collected sensor signals need to be acquired through the approach of the fast ensemble empirical mode decomposition (FEEMD) based Hilbert spectral analysis. Then by analyzing and extracting the peak phase shift information from the energy-time spectra, the time of arrival of excitation signals at both the actuator and the sensor can be captured and therefore the TOF for the group velocity estimation can be obtained. The detailed computation procedure of the damage index parameter is described in the ETPSM part, where it is illustrated that how the damage index TOF is calculated from the energy time spectrum. Some other methods have been also proposed and applied to the estimation of time of arrival for propagation waves using some algorithms, such as the threshold, peak-signal [19], double peak [20] and peak on-set time picking methods [18]. These methods pick the time of arrival based on the collected original signals and are relatively vulnerable to the uncertainty of signal noise levels. Our method determines the time of arrival based on the decomposed signals relying on FEEMD. The signals extracted by the decomposition contain the most significant components of original signals and are eliminated off noises, so that they are less oscillated and can facilitate more robust and evident searching for peak positions in the energy-time spectrum. The more advantages of the proposed method based on the FEEMD decomposition will be also supplemented in Section 4.1.

Once the group velocity is determined, the location of any damage can be inferred using the multi-damage index parameters—multi-functional multi-metrics. Accordingly, they are suitable for online structural health monitoring.

### 2.2. Rapid Multi-Damage Index Algorithm Based on Multi-Functional Multi-Metrics 

The proposed rapid multi-damage index algorithm (MDIA) is composed of two metrics that are the energy density metric and the energy time-phase shift metric. The rapid MDIA is then used to process the signal data from the sensor dynamic responses, infer the presence of any damage, determine the locations of damage, and diagnose the extents of damage. Meanwhile, the rapid MDIA is also shown to trace increasing amount of damage based on the multi-functional multi-metrics. It therefore has the potential to promote the development of damage visualization techniques. 

In order to interpret the benefits of this rapid multi-damage index algorithm, the fundamental concepts and formula with respect to the Hilbert transform and corresponding analytical signal processing methods are introduced as follows.

It supposes that a time domain signal s(t), and its Hibert transform H[s(t)] is defined as:

(2) $$H\left[s\left(t\right)\right]=y\left(t\right)=\frac{1}{\pi }\int_{-\infty }^{+\infty }\frac{s\left(u\right)}{t-u}du$$

The corresponding analytical signal z(t) originated from the signal s(t) can be expressed as:

(3) $$z\left(t\right)=s\left(t\right)+i\cdot y\left(t\right)=\lambda \left(t\right)e^{i\varphi \left(t\right)}$$

The benefit of the analytical signal z(t) obtained lies on the possibility to determine uniquely the genuine time-dependent variables. Then, there are the instantaneous parameters in Equation (3): λ(t) is defined as an envelope function describing the instantaneous amplitudes of the original signal s(t):

(4) $$\lambda \left(t\right)=\sqrt{s^{2}\left(t\right)+y^{2}\left(t\right)}$$

and φ(t) is defined as a phase function describing the instantaneous phase of the original signal s(t) *versus* time:

(5) $$\varphi \left(t\right)=\arctan\left(\frac{y\left(t\right)}{s\left(t\right)}\right)$$

For the multi-damage identification purpose, a sensor signal s(t) is first processed through the fast ensemble empirical mode decomposition so as to get the intrinsic mode functions (IMFs), which admit well-behaved Hilbert transform. 

#### 2.2.1. Fast Ensemble Empirical Mode Decomposition (FEEMD) and Hilbert Spectral Analysis 

**Figure 1:**
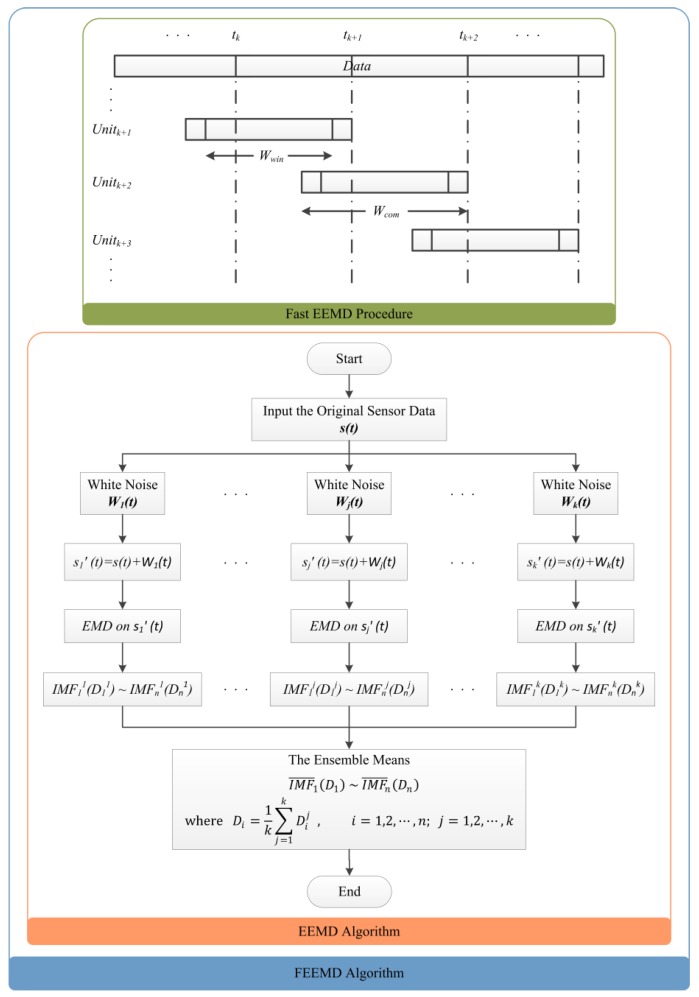
The fast ensemble empirical mode decomposition procedure.

The fast ensemble empirical mode decomposition is a new noise-assisted adaptive time-frequency data analysis method. It overcomes largely the scale (mode) mixing problem of the traditional empirical mode decomposition (EMD) [21] and provides physically unique decompositions. Essentially, it overcomes the main drawback of the traditional EMD when it is applied to data analysis with mixed and intermittent scales [22]. In the FEEMD decomposition process, the genuine intrinsic mode function (IMF) components are defined as the mean of an ensemble of trial divisions, and each trial consists of the decomposed signal plus a white noise of finite amplitude. By means of this ensemble mode, the scales can be separate clearly and naturally without any priori subjective criterion selection. Accordingly, the FEEMD becomes a truly dyadic filter bank for any sensor response data using the scale separation principle of the EMD. Through implanting finite noises, the FEEMD removes automatically the mode mixing problem in all cases. To clarify the computation and decomposition process, the flow chart in Figure 1 summarizes the major procedure of the FEEMD on a non-stationary sensor signal data recorded.

In the decomposition procedure of FEEMD, the original sensor response data are decomposed into a collection of intrinsic mode functions (IMFs). For instance, an original sensor signal s(t) is decomposed into n IMFs $D_{j}\left(t\right)$ and can be expressed as:

(6) $$s\left(t\right)=\overset{n}{\underset{j=1}{\sum }}D_{j}\left(t\right)+R_{n}\left(t\right)$$

where the residue $R_{n}\left(t\right)$ is a mean trend, from which no more IMF need to be extracted. It has been left out on purpose from the decomposition. From Equation (6), any given complex sensor data nonlinear or nonstationary can be decomposed into a set of simple intrinsic mode functions. IMFs are simple oscillatory functions and regarded as the basis of the expansion of the sensor data. As an effective IMF, it should satisfy the following conditions: (1) the number of extrema should be equal to the number of zero crossings; (2) the mean value of the envelopes defined by local extrema should be zero. To obtain IMF components $D_{j}\left(t\right)$, an iterative sifting procedure was implemented.

**Figure 2:**
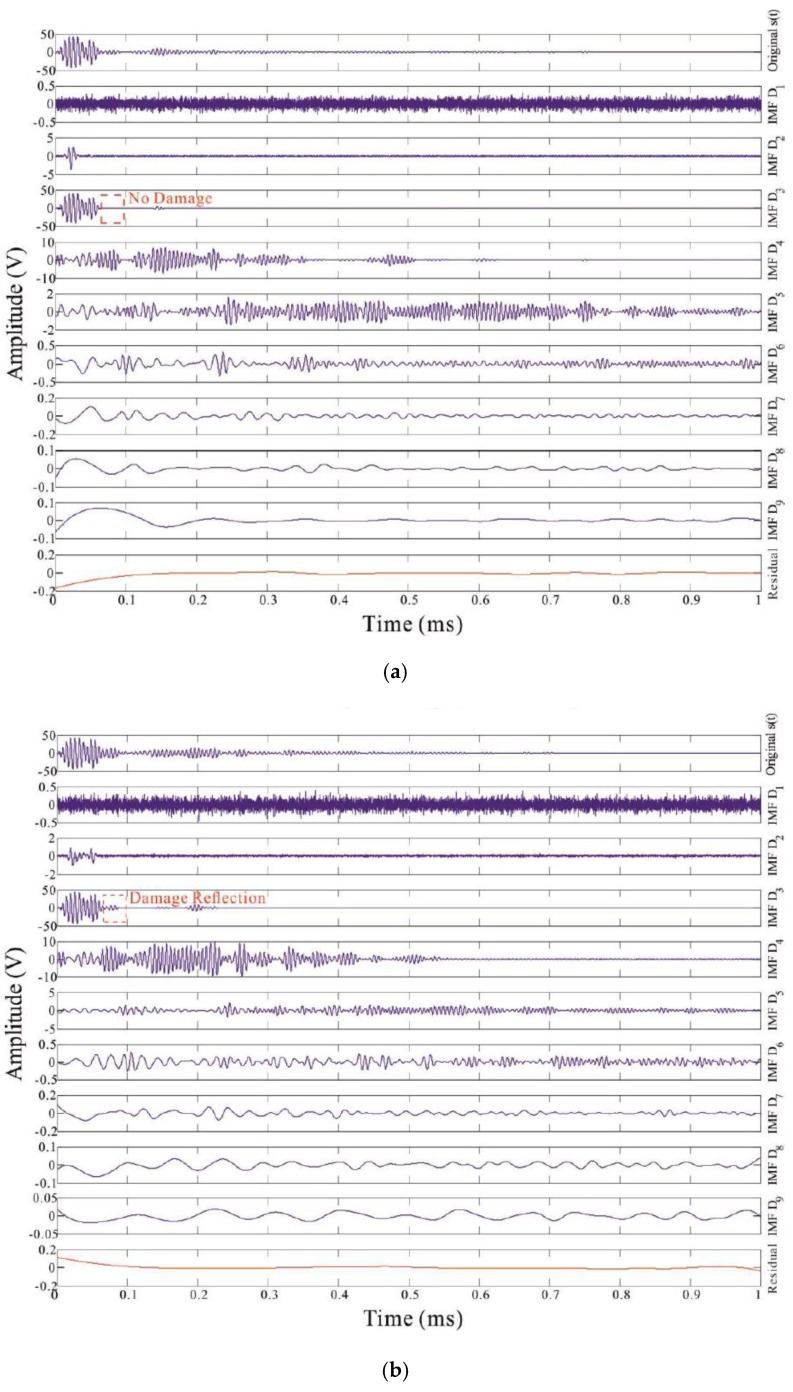
FEEMD processing for the sensor signals without (**a**), and with damage (**b**).

To interpret the advantage of the FEEMD, it is demonstrated that a case on non-damage and damage in an anisotropic composite panel structure. And the waveform from the excited diagnostic signal is made up of a 5-peaks narrow-band sine tone burst at 160 kHz modulated by a cosine Gaussian envelope. The decomposed IMFs resulting from the FEEMD processing on the selected sensor response signals are shown in Figure 2. 

Through FEEMD decomposition processing, any measured sensor data in time domain can be decomposed into *n* empirical modes. In other words, any sensor data from structural responses is mostly composed of different intrinsic modes of oscillation. As a result, each IMF is a frequency- and amplitude-modulated signal so that the IMFs have well-behaved Hilbert transformations. Then through the corresponding Hilbert transforming for the IMFs, any variation due to possible damage could be localized on the time as well as the frequency axis in the generated time-frequency spectrum. Meanwhile using the independent IMFs obtained from FEEMD decomposition, an evident full energy-time-frequency distribution for a given complex sensor data can be presented in the spectrum so that the local energy density and the instantaneous frequency can be obtained easily. In this way, with the property of time-dependent amplitudes and frequencies, each intrinsic mode function can therefore be treated as an individual signal, into which the Hilbert transform can be applied by:

(7) $$D_{j}\left(t\right)=\lambda_{j}\left(t\right)*\exp\left(i\int \text{​}\omega_{j}\left(t\right)dt\right)$$

where $D_{j}\left(t\right)$ is a monotonic component signal, which has a positive instantaneous frequency and a monotonically increasing phase. Actually in the Hilbert space, an original sensor data s(t) given can be expressed as the combination of each analytic signal corresponding to each IMF $D_{j}\left(t\right)$, or as a real part (RE) of the complex expansion:

(8) $$s\left(t\right)=\overset{n}{\underset{j=1}{\sum }}z_{j}\left(t\right)=RE\left[\overset{n}{\underset{j=1}{\sum }}\lambda_{j}\left(t\right)e^{i\omega_{j}\left(t\right)t}\right]$$

This approach of the FEEMD with Hilbert spectral analysis used in damage identifications is fundamentally different from the conventional Fourier transform method, of which the constant amplitude, frequency and other related information in frequency domain are derived from Fourier representations, as the instantaneous amplitude $\lambda_{j}\left(t\right)$ and angular frequency $\omega_{j}\left(t\right)$ in Equation (8) are both the functions of time *t*. Thus, a time-frequency spectrum can be obtained by the integration of H(t, ω) over the entire data period, and indicates the total energy contribution from each frequency value of the sensor data. In a word, the HSA-FEEMD method is suitable for complex data processing, especially in structural damage inspection applications, discovering tiny variation in oscillation during wave propagation. 

#### 2.2.2. Multi-Damage Index Parameters—Multi-functional Multi-Metrics (MFMMs) Based on HSA-FEEMD

The aim of the proposed multi-damage identification technique is to discover new damage index parameters, which can extract effectively and identify accurately complete multiple damage information. To realize an integrated damage inspection system, the different hierarchical goals of structural health monitoring (SHM) are classified, which include the presence of any damage, its location, its severity and the prediction of the remaining service life of a structure. From the measurement of a specific physical property of a monitored structure such as damping, stiffness, energy, *etc.*, a functional metric will become the key tool to give the necessary information about possible or diverse damaged situations. Thus, there are two defined multi-damage index parameters (MDIPs) used into the rapid multi-damage identification technique, which are the energy density metric and the energy time-phase shift metric. They are both robust and efficient functional metrics for multiple damage inspection in composite structures. 

##### Energy Density Metric (EDM)

A wave is an energy transport phenomenon. It travels through a medium such as a solid plate, and transports energy from one location (or its source) to another location without transporting matter. The amount of energy carried by a wave is thus related to the amplitude of the wave. Accordingly, the collected sensor signals are used to produce the corresponding energy time-frequency spectra. The energy time-frequency spectrum is defined as energy density metric that is obtained from the squared values of the instantaneous amplitudes. The amplitudes are deduced by the Hilbert transform of the high-energy IMFs. This metric is designated as a damage index parameter that can provide a high definition energy time-frequency representation. And also it can describe precisely the frequency content of any non-stationary or nonlinear signal based on the FEEMD process. Hence, the different features concealed in sensor signals can be revealed and better understood. In view of these reasons, an energy density spectrum needs to be found and used to identify and assess structural damage from the reflected wave energy. 

The energy density metric could not only quantify the extent of damage, but also map out the relationship between the released energy upon reflection due to damage and the defect growth through the corresponding energy time-frequency spectra from the sensor response data. Thus, in order to quantify the severity of any damage, a damage index parameter is introduced as a function of reflected energy in terms of the Hilbert time-frequency representation. The energy density is defined as a damage index parameter, which is expressed as Equation (9):

(9) $$E=\int_{\text{​}f}\int_{\text{​}t}\left|H_{e}\left(t,f\right)\right|=\int_{\text{​}f}\int_{\text{​}t}{\left|\lambda \left(t\right)\right|}^{2}$$

where $H_{e}\left(t,f\right)$ is called the Hilbert energy spectrum, λ(t) is the instantaneous amplitude resulting from the Hilbert transform. Since the amplitude and frequency of each decomposed signal with the FEEMD processing are both the functions of time, a three-dimensional time-frequency space [t, ω(t), λ(t)] can be produced. Then taking advantage of the characteristic of the three dimensional time-frequency spectrum, the instantaneous amplitude can be converted into the expression of energy to interpret any change of the propagating wave due to possible existed damage in the time-frequency spectrum. As Equation (9) shows, the transported energy is directly proportional to the square of the amplitude of the wave. Therefore, the energy density can be defined and represented in the Hilbert time-frequency spectrum. To reveal the EDM’s capability of multiple damage quantification and evaluation, the estimation results obtained by the energy density metric is illustrated in Section 4.2. The analysis of any damaged structure becomes facilitated by using the high-definition energy density spectrum. The reflection from damage is intuitively visible without any data pre-processing, which is indicated in Section 4.1. 

##### Energy Time-Phase Shift Metric (ETPSM)

**Figure 3:**
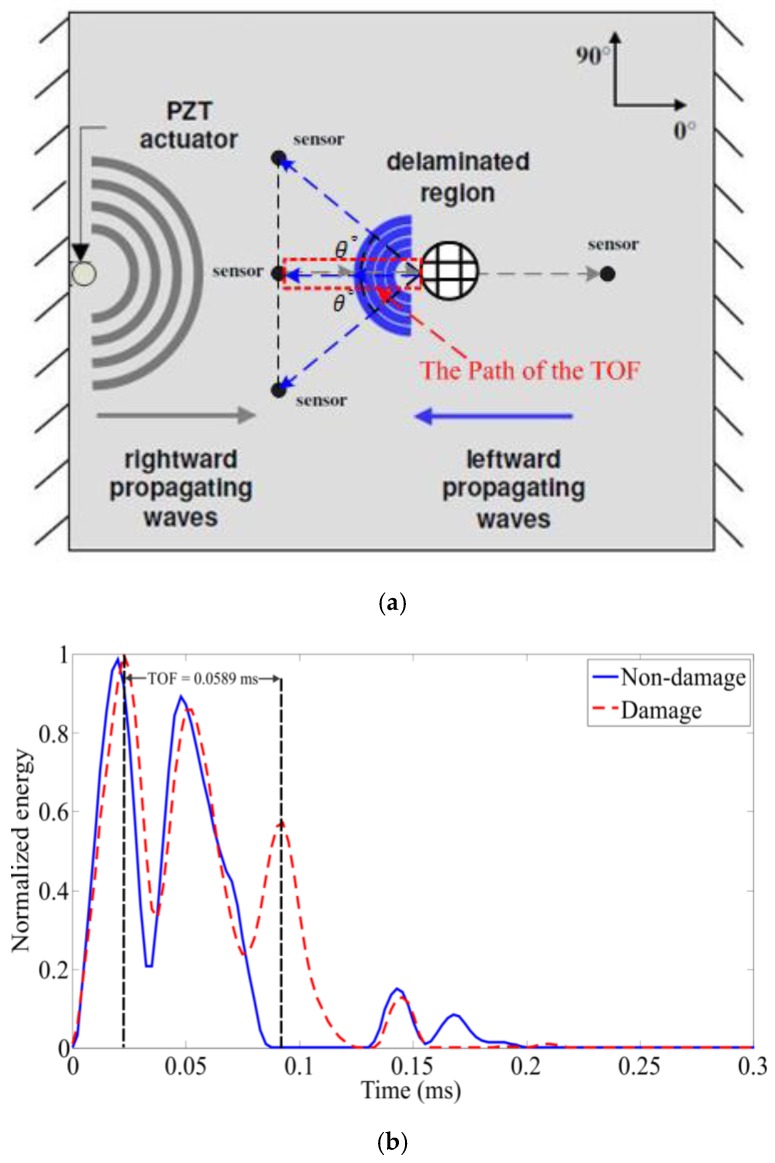
A demonstration of the TOF. (**a**) The wave propagating path encountering a delamination in a CFRP specimen; (**b**) Extraction of the TOF of the delamination.

In most damage detection schemes, the time of flight (TOF) is an important damage index parameter used often. The time of flight between the actuator and sensor on a structure is directly dependent on the layup structure of the composites, the properties of the standing (actuation) wave and the materials of the composites, which have been mentioned in Section 2.1. Here, the TOF is defined as the time duration taken from the energy peak of an actuation sensing wave to the energy peak of a reflected wave due to a damage, which is measured by a sensor. A demonstration of the TOF is illustrated in Figure 3. However, in order to easily obtain accurate time information to serve for the TOF, a high-definition energy time spectrum was proposed and developed based on the Hilbert spectral analysis. In the energy time spectrum, the time resolution become therefore more demarcative so as to conveniently calculate out a precise time of flight demanded, and consequently to obtain a precise position of the structural discontinuity with the information of the wave propagation velocity. 

The energy time-phase shift metric is proposed to locate any possible damage with an accurate determination of the time of flight, and to trace the severities of multiple damage by the phase shifts between the reflected waveforms. When a propagating wave encounters any damage, a part of the incident wavefront is reflected back while the rest diffracts through the damaged region. Actually, for the reflection wave, it is generated at the beginning of the damage. In this case, suppose that there are multiple damage with different sizes in a structure; the wave reflection from a bigger damage would be engendered sooner than that of a smaller damage located at the same position. Nevertheless, the scattered wave due to the existed damage can also be used to focus on the time of delay; thereby the different times between damaged and undamaged conditions are found and used in order to locate the damage.

Therefore, the time of flight between an actuation waveform and a reflection waveform due to damage can be precisely extracted from an energy time spectrum provided by the FEEMD based Hilbert spectral analysis method. In the energy time spectrum obtained from the selected IMF components containing the highest energies, the peaks give the arrival times of interest for the waves. Hence, the locations of various damage can be determined through the basic formula (10), which is defined as the damage positioning function (DPF):

(10) $$L_{dam}=\frac{C_{g}\left(\theta \right)\times t_{f}}{2}$$

where $L_{dam}$ is the distance of any damage away from a specific sensor. $C_{g}\left(\theta \right)$ is the group velocity of a propagating path. $t_{f}$ is the corresponding time of flight.

If the time resolution of an energy time spectrum obtained from the HSA-FEEMD processing is distinct enough to recognize the small fluctuations from the different times of flight corresponding to the diverse damage dimensions, then the estimation for the severities of progressive damage would be feasible. In other words, it would be possible to trace damage increasing even if the dimension of an initial damage is unknown.

## 3. Experimental Setup

**Figure 4:**
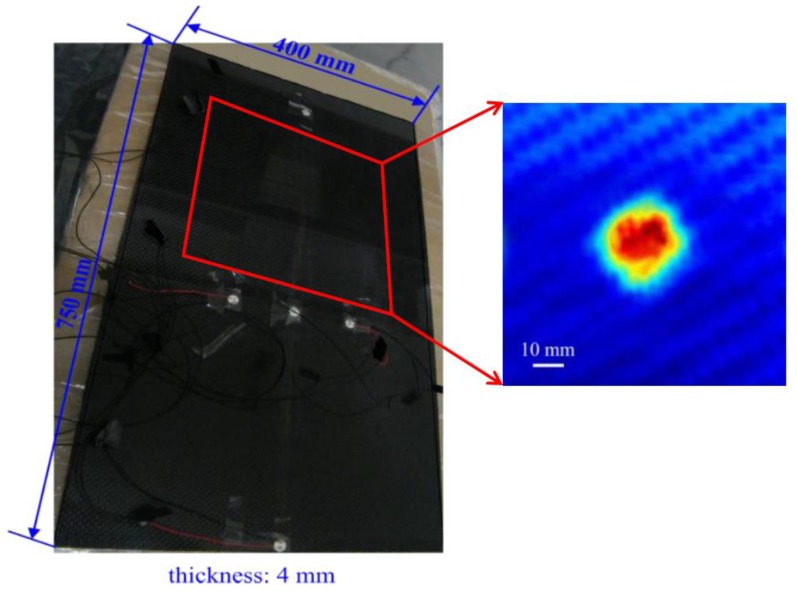
An instrumented composite specimen with a seeded delamination of 19 mm diameter. (**Left**) The instrumented composite panel with a seeded delamination. (**Right**) A zoomed-in ultrasonic phased image of a delamination region.

To effectively verify the multi-damage inspection capability of the rapid multi-damage identification technique, another material patch that differed from the carbon fiber prepreg laminate was used to seed as the delamination in the tested CFRP specimens. Accordingly, every delamination was made up of a circular Teflon patch and was seeded in the middle of a composite panel, which is located between a 90° and a 0° ply. Nevertheless, three delamination patches with different diameters of 10, 19 and 30 mm were produced and respectively seeded with progressive growth in another composite specimen. An example of an instrumented composite specimen with a delamination of 19 mm diameter is demonstrated in Figure 4a, and a zoomed-in ultrasonic phased image for the delamination region is presented in Figure 4b, which is captured partially from the full-scaled phased image of the ultrasonic scan. 

The tested composite plates were fabricated from CFRP laminates (T700S/E022) with the stacking sequence of $\left(0{}^{\circ}/90{}^{\circ}\right)/{\left[0{}^{\circ}/90{}^{\circ}\right]}_{s}/\;\left(0{}^{\circ}/90{}^{\circ}\right)$. The plates are all 750 mm long, 400 mm wide and 4 mm thick. They were instrumented with a designed PZT sensor array and a piezoceramic actuator. A circular PZT actuator with the diameter of 6 mm was bonded on the surface of the composite laminate at the bottom edge and was used to excite the tested structures with interrogating Lamb waves.

The actuation element creates a moment on the plate. This moment loading produces bending waves which are used for the multi-damage inspection. Most sensors of the designed PZT sensor array were bonded in the middle between the actuator and the delamination, and one sensor of the array was laid out in the behind of the delamination. The sensor array contained four circular sensor elements with a diameter of 6 mm for each. Because a sensor’s size is small enough, the influence of the sensors’ reflection can be considered negligible in the analysis and estimation of the collected signals. Meanwhile, the minimum size of which a probing wave is sensitive to a discontinuity is 10 mm. Thus, the propagation of an incident wave including wave velocity, propagation direction and so on isn’t affected by the sensors, most of the rightward propagation waves all diffract across the sensors, because a sensor’s size is much less than the minimal sensitivity size. Secondly, due to the delaminations seeded in the middle of the tested composite specimen, they didn’t emerge in the surface of the panel. As the laid sensors were affixed on the surface of the panel, wave propagation should not be affected much with the consideration of the boundary condition when the Lamb waves were actuated by the actuator. This will be proven by the experimental validations as well. 

A set of 5-peaks narrow-band sine tone burst signals with an actuation mode of the sweep frequencies from 60 kHz to 200 kHz and the amplitude of 50 V were used to excite the actuator. The sweep interval is 20 kHz. Meanwhile, the resulting response signals were observed and recorded at each sensor location. 

**Figure 5:**
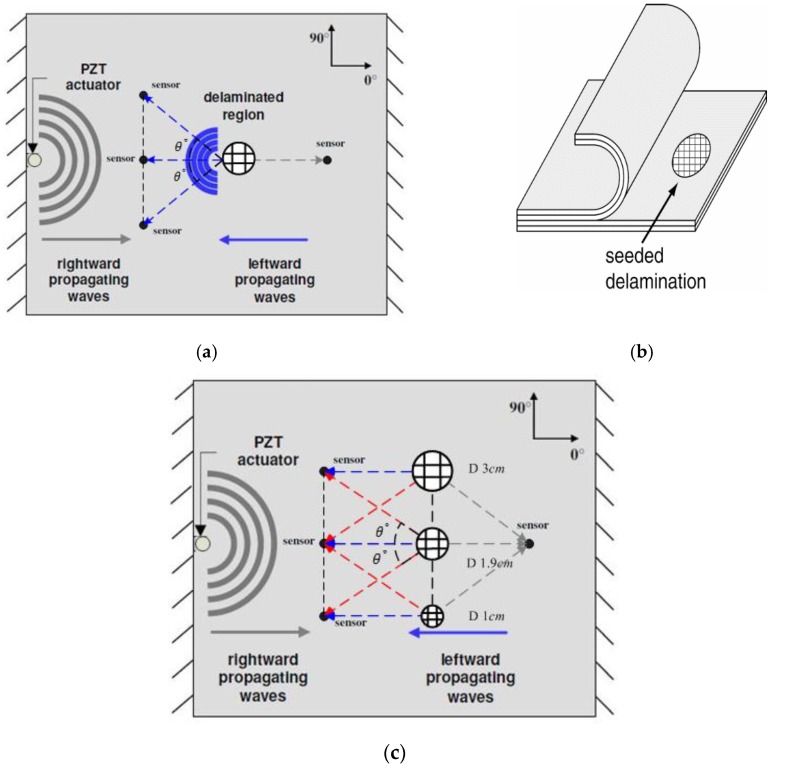
Experimental setups for the single and multiple damage inspections. (**a**) The inspection of single delamination in a CFRP specimen; (**b**) Teflon patches used to seed the delaminations; (**c**) The inspection of multiple delaminations in another CFRP specimen.

An instrumented composite specimen with the multiple delaminations that have separately 10, 19 and 30 mm diameters and another instrumented specimen with a single delamination of 19 mm diameter were examined by means of acousto-ultrasonics. The schematic diagrams of the experimental setup are illustrated in Figure 5. 

Figure 5a,b presents the frameworks of the two damage inspection cases, respectively: (1) the inspection of a single delamination seeded in a CFRP specimen and (2) the inspection case of multiple delaminations in the other CFRP panel, where the three delaminations of progressive diameters were seeded in the same layer and their center points were in a same vertical line. The delaminations invisible to the naked eyes were detected in the leftward propagating waves as well as in the selected sensor responses. 

**Figure 6:**
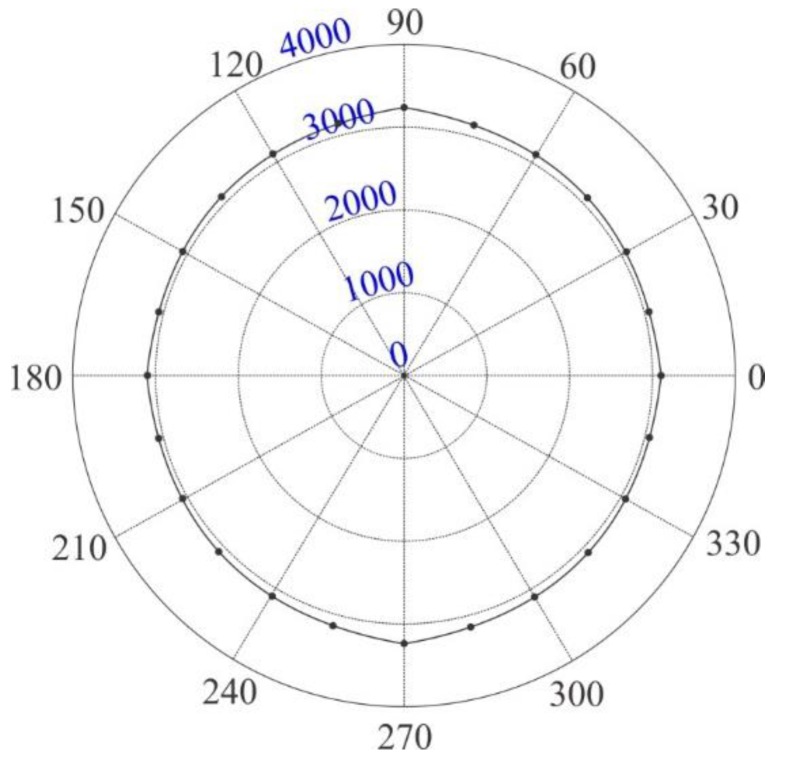
A map of group velocity variations depending on the propagation angles.

For the purpose of identifying complicated multi-damage, one needs to be aware of the angular dependence of group velocity in order to accurately estimate the locations of the multiple delaminations in the composite panel. Hence, a variation of group velocity in a map of propagation angles needs to be obtained by evaluating the time of flight of the Lamb wave propagating along different directions. Figure 6 shows that the mean value of the group velocity of the fundamental antisymmetric $A_{0}$ Lamb mode varies with the variation of the propagation angles. Divergences of wave group velocities due to the change of propagation angles are not observably apparent for the tested anisotropic specimen, because the propagation behavior of the fundamental antisymmetric Lamb wave in a cross-ply laminate is not only dependent on the propagation directions, but also dependent on the out-of-plane shear modulus of the material.

## 4. Results and Discussion

The seeded delaminations that were simulated by Teflon patches and introduced between two adjacent plies were successfully detected and quantified by the developed multi-functional multi-metrics. This section presents a series of damage diagnosis results from the characterized multi-functional multi-metrics using the proposed RMDI technique to demonstrate a robust and efficient multi-damage inspection scheme. From the measurement of a specific physical property (typically the variation of energy or VoE) of wave propagation in a structure, the energy density metric will give the necessary information to quantify the severities of various damage through the relevant energy density spectra. The energy time-phase shift metric is known as the key tool to infer the locations and sizes of multiple damage through the relevant energy-time spectra. Moreover, the increasing damage were shown to follow a linear trend obtained by the calculated prediction trend curves from the MFMMs, which can predict the extents of damage based on the released energies from the reflection waves. The details will be described in the following results and discussion. 

### 4.1. Determination of Multi-Damage by Transient Analysis Based on FEEMD

**Figure 7:**
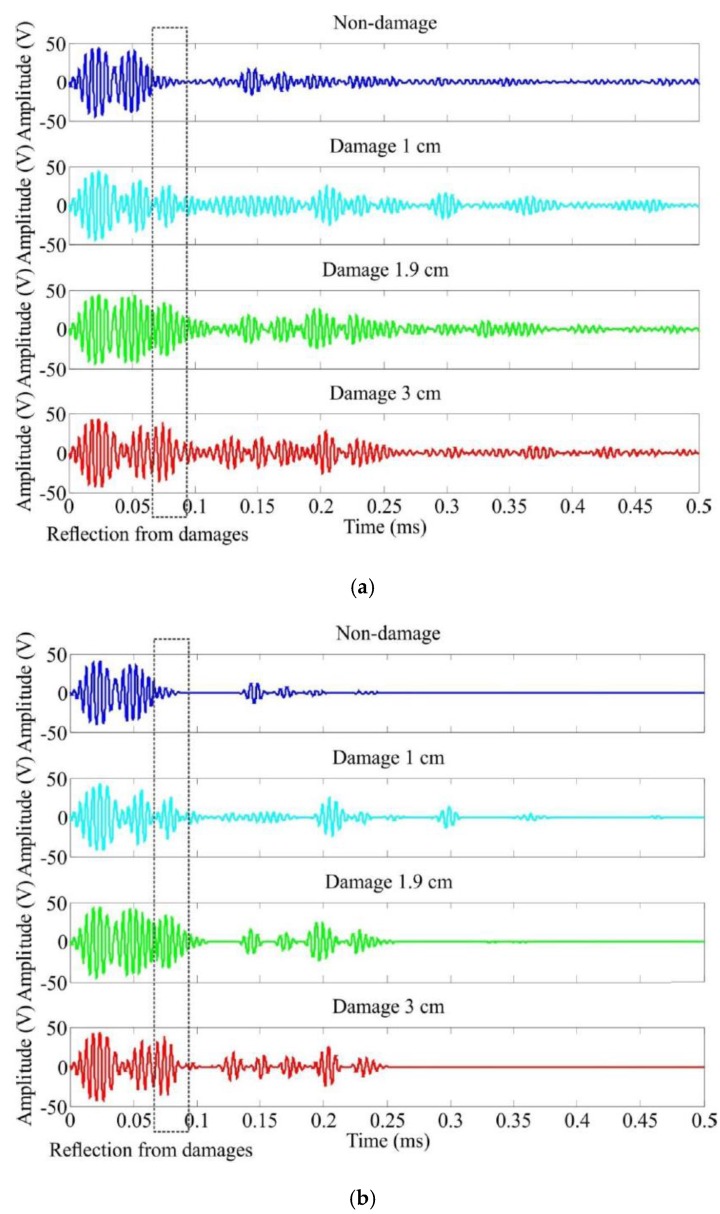
Multi-damage inspection comparison between two transient analysis methods (**a**) The conventional baseline method based on original signals; (**b**) The new proposed baseline method based on IMFs.

**Figure 8:**
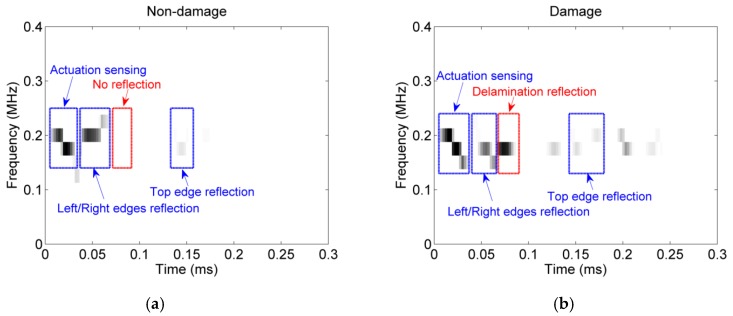
Energy density spectra for damage identification. (**a**) Non-damage condition; (**b**) A damaged condition—delamination presence.

To diagnose damage presence, it can be determined rapidly through the two approaches from the proposed RMDI technique:

(1) A new baseline method that is a direct appraisal approach based on the comparison results between the undamaged baseline and the various damage-lines that are all the intrinsic mode function components with the highest energies resulting from the fast EEMD process. The key advantage of the new baseline method is that it is more robust and discernible than the conventional baseline method [23,24,25,26] based on the comparison between the original undamaged and damaged signals. The comparison between the new and conventional baseline methods is illustrated in Figure 7. The other significant advantage of the IMFs based baseline method developed can provide a basis to obtain the unique time evolution of a structural response signal, which can be further used for energy density and instantaneous phase analysis. As shown in Figure 7, in the chosen time window, the response signals in Figure 7a are more oscillated than the IMF components in Figure 7b as they are the results of the fast ensemble empirical mode decomposition (FEEMD). 

(2) The energy density metric is able to quantify the variations of energies released from the reflections due to damage, and damage presence can be thus certified by the energy density metric, of which the demonstration is presented in Figure 8. The diagnosis results from the direct appraisal approach can also be verified by the energy density metric. The discussion of energy density metric will be detailed in Section 4.2. 

When a probing wave is excited, it propagates away from its origin. The energy carried by the wave will spread out in both space and time. Owing to dispersion and interaction with damage, the measured sensor signal is regarded as a combination of the excitation waveform versions that are scaled and shifted, as depicted in Figure 7. The incident waves from the actuator are located at 0.02 ms for each case whereas the reflections from the top or bottom edge arrive at 0.15 ms. A reflection waveform from the delamination should be comprised between the top/bottom edges reflection and the left/right edges reflection wave. A close observation of the wave signals does point to the presences of damage in the structures despite of slight differences. Although the reflections from the delaminations in the damaged composites may be weak and are probably drowned out by the boundaries reflections, the FEEMD processing is able to reveal those feeble reflections from delaminations if they exist in the wave signals.

### 4.2. Multi-Damage Quantification and Track by Energy Density Metric

**Figure 9:**
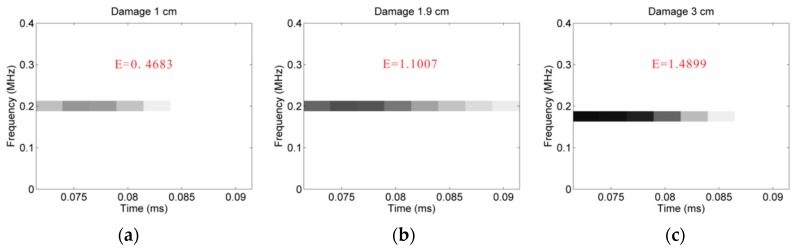
Energy density spectra for the different diameters of delaminations: (**a**) 1.0 cm; (**b**) 1.9 cm; (**c**) 3.0 cm.

As mentioned in the EDM part in Section 2.2.2, the energy density metric uses the high-resolution energy-time-frequency representation provided by the Hilbert spectral analysis to determine possible damage information. Thus, based on the energy density metric, a study of the three-dimensional images of both damaged and undamaged cases is carried out. From the estimated results calculated by the energy density metric shown in Figure 9, the energy released by the wave upon the damage growth increases with the size of the damage. 

**Figure 10:**
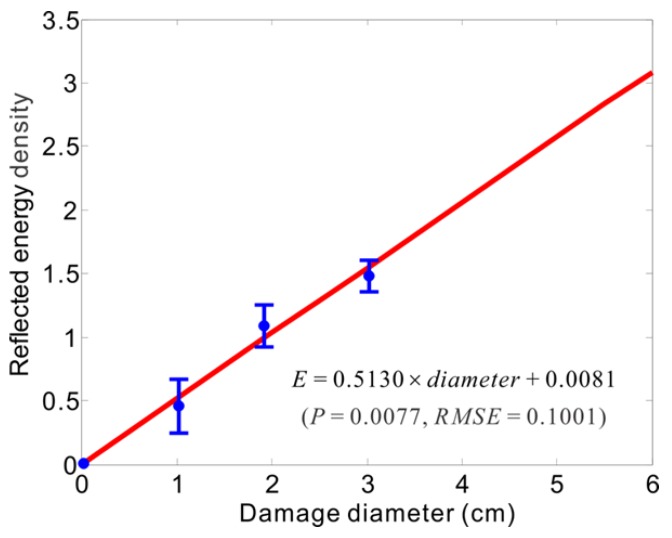
Prediction trend of multi-damage extents.

The energy increases monotonically with the diameter of damage as shown by the prediction trend in Figure 10. The prediction of damage sizes using the density estimation of released energies due to damage is therefore possible with the linear fit. The damage assessment (DA) method based on the damage prediction trend curve (DPTC) obtained from the average results of energy densities with the root-mean-square errors (RMSE) follows the linear fit between damage diameters and energy densities through the several validation tests for the three progressive delaminations, which is presented in Figure 10. Meanwhile, to verify the prediction accuracy of the energy density based DPTC, a new set of damage validation tests are necessary to be implemented, and the energy density value of any unknown damage is determined firstly using the EDS, subsequently the unknown damage can be evaluated through the linear prediction representation found. The average error ratio between the estimated damage and actual damage is 8.6%. 

As shown in Figure 10, two regression parameters, which are the P-value of Student’s t-test and the RMSE, are calculated to evaluate the reliability of the linear damage prediction trend curve. As the prediction curve does describe the relationship between the reflected energy density and the damage extent from Figure 10, the P value thus needs to be calculated and is equal to 0.0077, falling within the confidence range (P < 0.05), and RMSE = 0.1001. Accordingly, the obtained linear DPTC is sufficient to the demand of damage prediction. From the above multi-damage identification results, the energy density metric demonstrates its powerful competence to quantify damage by its damage index parameter that is the energy density value, and to trace increasing damage by the damage prediction trend curve. 

### 4.3. Multi-Damage Localization and Track by Energy Time-Phase Shift Metric

Accurate identification of localized events is required to determine the locations of various damages in a structure precisely and to quantify the defect growth. Then, in order to determine damage locations, this can be implemented rapidly by the damage positioning function Equation (10) based on the energy time-phase shift metric (ETPSM), where an estimation of the time interval between the actuation sensing wave and reflection waveform can be determined precisely from the high time resolution energy-time spectrum transformed. 

Hence, the importance of the determination of the time of flight between the actuation sensing wave and reflection waveform has been in evidence as described in the previous ETPSM part in Section 2.2.2. The developed phase shift metric uses the high time resolution of the FEEMD-based Hilbert transform to extract the accurate time of flight so as to locate any possible damage and to trace the increasing damage in a composite structure. 

**Figure 11:**
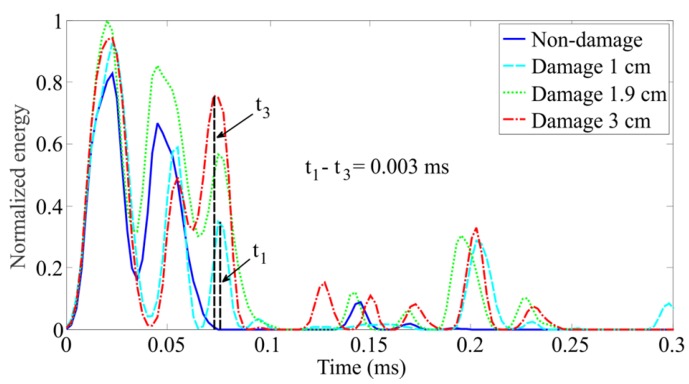
Energy time spectrum for the phase shifts of progressive damage.

The best method to obtain the interested time of flight is to look for the reflected energy peaks of damage for each IMF component containing the highest energy from the sensed signals’ FEEMD decompositions in the energy-time spectrum. In the case of three different delaminations, the energy-time spectrum for each IMF signal with the highest energy is plotted in Figure 11. Actually, the energy time-phase shift metric also meets a challenge that the reflections from boundaries mix with the reflections from the delaminations. 

The issue of the reflections from the left/right and top boundaries had been discussed and addressed in Section 4.1, of which two estimation results have been shown in Figure 8. Although the boundary reflections may increase the difficulty of damage inspection and identification, they don’t affect the implement of the developed ETPSM to damage identifications. The proposed ETPSM can be applied in a more extensive condition as the time of arrival (TOA) for a delamination is determined by the peak of the reflection energy of a probing wave using the Hilbert spectral analysis, rather than the direct information of wave packets. If the peaks of the two energy reflections during the wave propagation can be distinguished, the time of arrival of each energy reflection can be evaluated and thereby the corresponding damage can be located and identified. Since the reflected energy is defined as the overall energy accumulated within the time period of full width at half maximum (FWHM) of the energy peak, the reflected energy from a delamination may be overestimated if the wave reflections from diverse discontinuities or wave packets overlapped excessively and therefore the estimation of damage dimension may not be accurate either. In this case, Gaussian curves are used to fit the energy curves of peaks and each reflected energy is approximated as the integration of the Gaussian function over the period of full width at half maximum of the Gaussian peak. In summary, the ETPSM proposed in this study can also be applied in the scenario of overlapping wave packets case. However, the ETPSM may not work in the case when two peaks of the energy reflections completely overlap. The minimum time resolution of two wave peaks that can be differentiated is around 5.7 µs, which corresponds to 1 cm in wave propagation distance. The dead zone is defined as the region within 1 cm from a structural boundary. However, in real engineering cases, there actually may not exist damage within the range of 1 cm from a structural boundary. The quantification of increasing damage is also possible using the proposed damage assessment (DA) method. The DA method is based on the energy time-phase shift metric (ETPSM) for multi-damage inspection in composite structures. 

**Figure 12:**
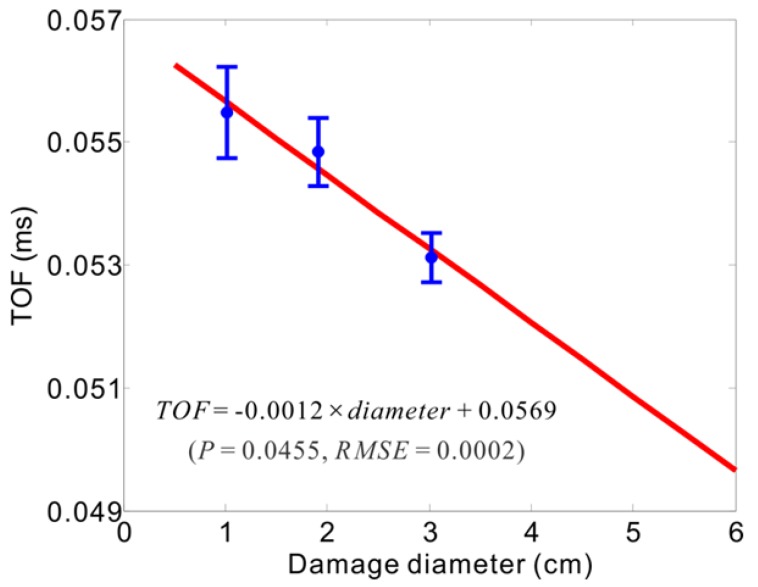
Prediction trend on progressive damage.

Once the relevant energy time-phase shift metric was computed and obtained using a new set of the sensed data measured from the damage tests, the amount of any new damage unknown can be inferred and its extent can be estimated further through the ETPSM combined with the TOF based DPTC. For instance, through the ETPSM obtained, the TOF of an unknown damage can be determined, of which is 0.0531 ms. Subsequently, the diameter of this unknown damage can be estimated by the TOF based DPTC presented in Figure 12. The estimated diameter of the unknown damage is of 3.10 cm, of which the estimation error is given as 3.4% in contrast to its actual diameter of 3 cm. 

In Figure 12, the TOF based damage prediction trend curve is obtained from the average TOF results of damage with the root-mean-square errors (RMSE) through the several validation tests for the three progressive delaminations. The DPTC follows the linear fit between damage diameters and TOF values from the ETPSM. To verify the prediction accuracy of the TOF based DPTC, a new set of sensed data with multi-damage information were used, and the efficient TOF values were are extracted and determined from the ETPSM, finally any unknown damage in a composite structure can be evaluated through the linear prediction representation found. The average error ratio between the evaluated damage and actual damage is 9.2%.

For the TOF-based damage prediction trend curve, the P-value and the RMSE for the regression are estimated to be 0.0455 in the confidence range P < 0.05 and 0.0002 ms, respectively. Basically, the TOF-based DPTC does satisfy the demands of damage prediction. From the average estimation error ratio, the prediction accuracy of the ETPSM based DA method may be qualified to practical multi-damage quantification applications. 

## 5. Conclusions

In this paper, the combination of the CLPT theory and finite element modelling is adopted to model the dynamics of the laminated composites. Since Lamb waves are used to excite the structures, the wavenumber-frequency relationships of Lamb wave propagation in laminated composites are determined and computed. As a result, the wave group velocity of the $A_{0}$ mode from Lamb wave propagation can be determined. The rapid multi-damage identification technique as an effective damage inspection and assessment tool is used to process the intricate signal data, and to infer the presence of any damage, and to determine the locations of damage that are not visible in the visual inspection, and to quantify and trace various extents of damage:

1. The energy density metric from the RMDI technique can rapidly quantify various amounts of damage by its damage index parameter (DIP)—the energy density values.
2. The energy time-phase shift metric from the RMDI technique can locate various damages by its DIP—the time of flight (TOF) from the high-time resolution spectra in real time mode.
3. Furthermore, the RMDI technique also demonstrates its powerful competence to trace increasing damage. For various extents of damage in composite structures, the two metrics can both be used to track the progressive damage by their individual damage prediction trends. The energy density metric can track multiple damage by its damage-energy variation curve. The energy time-phase shift metric can trace increasing damage by its damage-time difference curve derived from the defined damage prediction function (DPF).

To sum up, through all of the designed damage experiments, the rapid multi-damage identification technique based on the fast ensemble empirical mode decomposition with its associated Hilbert spectral analysis has shown promising results in the analysis of time-series data. Also, the rapid multi-damage identification technique evidently indicates a potential as an online onboard damage inspection and assessment tool. This great potential could be applied to facilitate the diagnosis and prognosis for the health or safety states of aerospace structures.
